# Pre-gestational stress reduces the ratio of 5-HIAA to 5-HT and the expression of 5-HT1A receptor and serotonin transporter in the brain of foetal rat

**DOI:** 10.1186/1471-2202-13-22

**Published:** 2012-02-28

**Authors:** Yuejun Huang, Hongwu Xu, Hui Li, Hanhua Yang, Yunbin Chen, Xuechuan Shi

**Affiliations:** 1 Department of Pediatrics, Second Affiliated Hospital of Medical College of Shantou University, North Dongxia Rd, Shantou 515041, Guangdong, China; 2 Department of Neurosurgery, Second Affiliated Hospital of Medical College of Shantou University, North Dongxia Rd, Shantou 515041, Guangdong, China; 3 Mental Health Center of Shantou University, Tai Shan Rd, Shantou 515041, Guangdong, China; 4 Maternal and Child Health Hospital of Guangdong Province, West Guangyuan Rd, GuangZhou 510010, Guangdong, China

**Keywords:** Corticosterone, HPA axis, Serotonin, Serotonin transporter, Stress

## Abstract

**Background:**

Many studies have found that stress before or during pregnancy is linked to an increased incidence of behavioural disorders in offspring. However, few studies have investigated hypothalamic-pituitary-adrenal (HPA) axis activity and the serotonergic system as a consequence of pregestational stress. In the present study, we investigated the effect of pre-gestational stress on HPA axis activity in maternal rats and their foetuses and examined whether changes in HPA axis activity of maternal rats produced functional changes in the serotonergic system in the brain of foetuses.

**Results:**

We used the behavioural tests to assess the model of chronic unpredictable stress (CUS) in maternal rats. We found the activity in the open field and sucrose consumption was lower for rats with CUS than for the controls. Body weight but not brain weight was higher for control foetuses than those from the CUS group. Serum corticosterone and corticotrophin-releasing hormone levels were significantly higher for mothers with CUS before pregnancy and their foetuses than for the controls. Levels of 5-hydroxytryptamine (5-HT) were higher in the hippocampus and hypothalamus of foetuses in the CUS group than in the controls, and 5-hydroxyindoleacetic acid (5-HIAA) levels were lower in the hippocampus in foetuses in the CUS group than in the control group. Levels of 5-HIAA in the hypothalamus did not differ between foetuses in the CUS group and in the control group. The ratio of 5-HIAA to 5-HT was significantly lower for foetuses in the CUS group than in the control group. Levels of 5-HT1A receptor were significantly lower in the foetal hippocampus in the CUS group than in the control group, with no significant difference in the hypothalamus. The levels of serotonin transporter (SERT) were lower in both the foetal hippocampus and foetal hypothalamus in the CUS group than in the control group.

**Conclusions:**

Our data demonstrate that pre-gestational stress alters HPA axis activity in maternal rats and their foetuses, which is associated with functional changes in 5-HT activity (5-HT, 5-HIAA and ratio of 5-HIAA to 5-HT), as well as the levels of the 5-HT1A receptor and SERT in the hippocampus and hypothalamus of foetuses.

## Background

Many studies in humans and animals have found that emotional disturbance and distress before or during pregnancy that results from natural or human-made disasters, chronic interpersonal tension or adverse conditions in the home or workplace are linked to an increased incidence of behavioural disorders in offspring [1-3]. These disorders include impaired memory and language development, autism, attention deficits, schizophrenia, anxiety disorder and depression.

Studies of the neurobiological mechanisms underlying the interaction between maternal stress and adult mental disorders suggest the involvement of multiple neurotransmitter systems [4,5]. Changes in activity of the central serotonin [5-hydroxytryptamine (5-HT)] system play a major role in many of these behavioural aberrations [6,7]. During pregnancy, the 5-HT system has a fundamental role in the development of the central nervous system of the foetus, and 5-HT neurotransmission is involved in the activation and feedback of the hypothalamic-pituitary-adrenal (HPA) axis throughout life [8,9]. Because serotonin levels within the synaptic cleft are regulated by the activity of the serotonin transporter (SERT) protein, SERT is critical for regulating serotonergic function. Stressful experiences and changes in the SERT gene or protein levels have been linked to behavioural disorders [10-12]. For example, genetic variants in the SERT gene have been associated with increased anxiety [13,14], neuroticism and depressive symptoms; SERT-knockout mice show altered levels of 5-HT1A receptors and a stress-mediated increase in corticosterone (COR) and corticotrophin-releasing hormone (CRH) [15]. Alterations in SERT function can cause adaptive changes in 5-HT1A receptor levels [16-18]. In addition, humans with depression and decreased SERT levels showed changes in 5-HT1A receptor density [19]; changes in 5-HT1A receptor function were noted in SERT-knockout mice [18]. However, these studies have focused on the effects on offspring of stress exposure during pregnancy. Only a few studies have examined the effect of chronic unpredictable stress (CUS) before pregnancy on neurobiological variables in offspring [3].

To date, no studies have investigated HPA axis activity or the serotonergic system in corresponding brain regions of the foetus as a consequence of pre-gestational stress. Therefore, the first goal of this study was to investigate whether pre-gestational stress alters HPA axis activity in maternal rats and their foetuses. Changes in the HPA-axis activity of maternal rats may not necessarily produce changes in the 5-HT system of the foetus, so the second goal of this study was to examine whether changes in the HPA axis activity of maternal rats produced functional changes by measuring 5-HT activity [5-HT, its metabolite 5-hydroxyindoleacetic acid (5-HIAA) and the ratio of 5-HIAA to 5-HT], as well as 5-HT1A receptor and SERT levels in corresponding brain regions of the foetal brain. And the third goal of this study was to investigate the mechanisms involved.

## Methods

### Animals

Adult male (n = 10) and female (n = 20) Sprague-Dawley rats (approximately 8 weeks old; 250-300 g and 200-250 g, respectively) were provided by the Animal Center of Shantou University. Female rats were nulliparous. Before the beginning of any stressful procedures, female rats were divided into 2 groups for treatment: control (n = 8) and chronic unpredictable stress (CUS) (n = 12). Female rats were housed singly, and male rats were housed in pairs in plastic, non-transparent cages (60 × 40 × 25 cm) in separate rooms under controlled 12-h light/12-h dark conditions (lights on at 08:00) and temperature (24°C). All rats had free access to food and water throughout the experiments, unless specified by the experimental procedure.

Female rats exhibited a normal estrous cycle (4-5 days of estrous) before the start of the experiment, with equal distribution of different stages of the estrous cycle. The estrous cycle was reconfirmed during the last week of CUS with the use of vaginal smears [20].

All animal experiments were reviewed and approved by the Medical Animals Care and Welfare Committee of Shantou University Medical College (Shantou, China). All studies were carried out in accordance with the US National Institutes of Health Guide for the Care and Use of Laboratory Animals (NIH Publications No. 80-23 revised 1996). Every effort was made to minimize the number of animals used and reduce suffering.

### CUS procedure

Body weight of females was measured before the start of CUS (W0) and once a week (W1, W2, W3) during CUS. The CUS procedure and sucrose intake test and open field test were as we previously described [3]. The schedule is in Table 1 and Table 2.

**Table 1:** The adaptation period of sucrose intake test

Time	Course of the experiment
The 1st day 8:00	food and water deprivation for 24 h

The 2nd day 8:00 an interval of 3 days	one-bottled-sucrose test sessions (last 1 h)

The 6th day 8:00	food and water deprivation for 24 h

The 7th day 8:00	one-bottled-sucrose test sessions (last 1 h)

an interval of 3 days	

The 11th day 8:00	food and water deprivation for 24 h

The 12th day 8:00 an interval of 3 days	one-bottled-sucrose test sessions (last 1 h)

The 16th day 6:30	weighed body weight and open field test

8:00	food and water deprivation for 24 h

The 17th day 8:00	one-bottled-sucrose test sessions (last 1 h)

The 17th day 9:00	The beginning of CUS procedure

**Table 2:** The CUS procedure

Time	Course of the experiment
The 1st day 9:00	cage rocking (5 times per second) for 15 min

The 2nd day 9:00	swimming in cold (4°C) water for 5 min

The 3rd day 9:00	soiled cage (250 ml of tap water into the sawdust bedding) for 24 h

The 4th day 9:00	lights on overnight

The 5th day 9:00	cage tilting for 24 h

The 6th day 9:00	restraint for 12 h

The 7th day 8:00	food and water deprivation for 24 h

The 8th day 8:00	one-bottled-sucrose test sessions (last 1 h)

9:00	soiled cage for 24 h

The 9th day 9:00	electric stimulus (1.0 mA, each time for 1 second, 10 times per minute) for 5 min

The 10th day 9:00	elevated temperature (40°C) for 15 min

The 11th day 9:00	cage rocking for 15 min

The 12th day 9:00	lights on overnight

The 13th day 9:00	restraint for 12 h

The 14th day 8:00	food and water deprivation for 24 h

The 15th day 8:00	one-bottled-sucrose test sessions (last 1 h)

9:00	lights on overnight

The 16th day 9:00	swimming in cold water for 5 min

The 17th day 9:00	restraint for 12 h

The 18th day 9:00	electric stimulus for 5 min

The 19th day 9:00	cage rocking for 15 min

The 20th day 9:00	soiled cage for 24 h

The 21th day 9:00	cage tilting for 24 h (The CUS procedure finish)

The 22th day 6:30	weighed body weight and open field test

8:00	food and water deprivation for 24 h

The 23th day 8:00	one-bottled-sucrose test sessions (last 1 h)

### Pregnancy of female rats

When the CUS procedure was finished (24 h after the last stressor), all female rats were housed in pairs with 1 male for 1 week for mating. The day when sperm was observed in vaginal smears was designated as embryonic day 0 (E0), and blood samples were taken from the vena cava caudalis between 10:00 and 12:00. Nest material was provided for gestational female rats, which were housed singly and not disturbed.

### Blood and brain tissue samples of foetuses

All control rats (n = 8) and 9 of 12 CUS rats became pregnant. All pregnant rats were still pregnant at embryonic day 20 (E20). The pregnant rats were anaesthetised with pentobarbital. The uteri of pregnant rats were exposed by opening the peritoneal cavity after anaesthesia. Then the foetuses were dislodged from the uterus and weighed. There were 87 foetuses (47 females and 40 males) from the pregnant rats of the control group and 92 foetuses (43 females and 49 males) from the CUS group. To assess serum COR and CRH levels, blood samples were quickly taken by removing the head of the foetus. Due to the low volume of serum in foetuses, COR and CRH levels were measured in pooled serum samples from foetuses from the same pregnant rat. All blood samples were collected between 10:00 and 12:00. The brains of foetuses were rapidly removed and weighed. Each hypothalamus was dissected on ice into two equal parts divided by the midline. The left side of hippocampus and one of the equal part of hypothalamus were homogenized and deproteinized in 500 μl of 0.2 N perchloric acid (Merck KgaA, Darmstadt, Germany) solution containing 7.9 mM Na_2_S_2_O_5 _and 1.3 mM disodium ethylenediaminetetraacetic acid (Na _2_EDTA) (both from Riedel-de Haën AG, Seelze, Germany). The homogenate was centrifuged at 14,000 rpm for 30 min in 4°C, and the supernatant was stored at - 80°C. Analysis involved high-performance liquid chromatography (HPLC) as described [21] with some modification. All samples were measured within 1 month after homogenization; previous studies have shown that monoamines remain stable up to 1 month after homogenization [22]. The right side of hippocampus and the other part of hypothalamus were frozen in liquid nitrogen and stored at -70°C for quantitative real-time reverse transcription polymerase chain reaction (RT-PCR) and western blot analysis. Before RT-PCR and western blot analysis, the brain tissue was pounded to pieces. Several of these pieces were used for RT-PCR, and the others were used for western blot analysis.

### Measurement of COR and CRH levels

Blood samples were centrifuged at 4000 rpm for 30 min. The serum was collected and frozen at -70°C until use. Serum COR and CRH levels were determined with a standard radioimmunoassay kit (ICN Biomedicals, Costa Mesa, CA, USA). The sensitivity of the COR radioimmunoassay kit was 25 pg/ml. The inter- and intra-assay coefficients of variation were both 8%. The sensitivity of the CRH radioimmunoassay kit was 40 pg/ml. The intra- and inter-assay variability were 3.84% and 10.36%, respectively.

### HPLC procedure

HPLC was used to assay 5-HT and 5-HIAA. The HPLC procedure was performed according to the method previously described [21] with some modifications. For monoamine analysis, an Agilent HC-C18 analytical column (250 mm × 4.6 mm, 5 μm; Agilent, USA) was used. The mobile phase consisted of 20% methanol and 80% aqueous solution, which contained 30 mM citric acid, 40 mM sodium acetate, 0.2 mM ethylenediaminetetraacetic acid (EDTA) disodium salt and 0.5 mM octanesulfonic acid sodium salt, at a flow rate of 1.0 ml/min and at pH value of 3.8. The level of 5-HT and 5-HIAA were detected using a Waters 474 scanning fluorescence detector (Waters, USA) with the excitation and emission wavelengths set at 280 nm and 330 nm, respectively. The HPLC system was connected to a computer to quantify all compounds by comparing the area under the peaks with the area of reference standards with specific HPLC software (Chromatography Station for Windows). The turnover ratio of 5-HIAA to 5-HT is considered an index of the activity of cells that cause release of 5-HT, re-uptake and metabolism to 5-HIAA [23].

### Quantitative real-time RT-PCR

Brain tissues were stored at -70°C until the assay for 5-HT1A mRNA level by quantitative real-time RT-PCR as described [24]. Briefly, RNA was isolated from tissue by the TRIzol method [25]. Samples were treated with DNase to remove contaminating DNA before cDNA synthesis by reverse transcription with the SuperScript II system (Invitrogen). The oligonucleotide primers used were for 5-HT1A (forward, 5'-CCAAAGAGCACCTTCCTCTG-3', reverse, 5'-TACCACCACCATCATCATCA-3'); GAPDH, as a housekeeping gene (forward, 5'-ACACTGTGCCCATCTACGAGG-3', reverse, 5'-AGGGGCCGGACTCGTCATACT-3'). PCR amplification of cDNA involved the Quantitect SYBR Green PCR kit (Qiagen). The products of PCR were monitored in real time by the MyiQ Detection System (Bio-Rad), and gene expression was determined by the 2_T_^-ΔΔC ^method [26].

### Western blot analysis

Brain tissues were homogenized in cell lysis buffer for western blot analysis (Beyotime, Jiangsu, China). Protein (50 μg/lane) separated by SDS-PAGE was blotted onto nitrocellulose (NC) membranes by electrophoretic transfer. Blots were incubated in blocking buffer, which was a mixture of 10% non-fat dry milk powder in tris buffered saline containing 0.5% Tween-20 (TBS-T) for 1 h at room temperature and washed three times for 10 min each in TBS-T. Blots were then incubated at 4°C with the primary antibodies mouse anti-β-actin or rabbit anti-SERT (both 1:1000 dilution; Cell Signaling, USA), and washed three times for 10 min each in TBS-T. Blots were incubated with the appropriate horseradish peroxidase-labelled secondary IgG antibody for 2 h at room temperature, washed three times for 10 min each in TBS-T, treated with BeyoECL reagents (Beyotime, Jiangsu, China), and exposed to film (Kodak, USA). Band intensity was quantified by use of Bandscan 5.0 (Glyko Bandscan software). The relative protein level of SERT was calculated relative to that of β-actin.

### Statistical analysis

Data are presented as mean ± S.D. The differences in body weight and sucrose intake between the two maternal groups in the same week were analysed using a *t*-test, and the effect of CUS on body weight and sucrose intake in maternal rats during the CUS procedure was analysed by one-way repeated ANOVA. The data in the open-field test were measured by *t *test. We performed gender-specific comparisons of foetuses. Data for foetuses were analyzed by *t *test. The correlations between COR or CRH levels and the ratio of 5-HIAA to 5-HT or 5-HT1A and SERT expression for foetuses were analyzed by Bivariate correlation analysis. Statistical significance was set at *P *< 0.05.

## Results

### Effect of CUS on body weight and behaviour of maternal rats

Table 3 shows that body weight and sucrose intake of the CUS group were lower than the body weight and sucrose intake of the control group at week 2 and week 3. One-way repeated ANOVA with CUS as the independent factor and time as the repeated factor revealed the effect of CUS on body weight [F(1,18) = 12.379,*P *= 0.02] and sucrose intake [F(1,18) = 9.214, *P *= 0.043] in maternal rats. The differences in relative sucrose intake were similar to those for sucrose intake and not presented here. The moving behaviour of CUS maternal rats was significantly lower at week 3 than at week 0 (see Table 4).

**Table 3:** Body weight and sucrose intake of maternal rats during CUS

Target	Groups	n	W0	W1	W2	W3
Body weight (g)	Control	8	237.3 ± 3.9	245 ± 2.7	252.3 ± 5.3	258.6 ± 5.8
			
			(234~240.5)	(242.8~247.2)	(247.9~256.6)	(253.8~263.5)
	
	CUS	12	237.6 ± 5.9	242.1 ± 7.4	245.8 ± 7**	249.5 ± 7.6**
			
			(233.9~241.3)	(237.4~246.8)	(241.3~250.2)	(244.7~254.3)

Sucrose intake (g)	Control	8	10.09 ± 1.05	10.5 ± 0.85	11.33 ± 1.53	12.1 ± 1.36
			
			(9.21~10.97)	(9.79~11.21)	(10.05~12.61)	(10.96~13.23)
	
	CUS	12	10.16 ± 0.97	10.4 ± 1.28	10.67 ± 1.66*	10.85 ± 1.8**
			
			(9.54~10.78)	(9.59~11.21)	(9.61~11.73)	(9.71~11.99)

Data are mean ± S.D. from week 0 to week 3. Data in bracket are 95% confidence intervals for each relevant statistic. *, **:*P *< 0.05, *P *< 0.01 compared with control by the same week

**Table 4:** Moving behaviour of maternal rats in open field test

Groups	n	Time	Total path (cm)	Central path (cm)	Peripheral path (cm)
Control	8	W0	2925.4 ± 254.1	293.3 ± 84.3	2632.1 ± 182.
			
			(2713.0~3137.8)	(222.8~363.8)	(222.6~336.6)
	
		W3	2861.1 ± 283.8	279.6 ± 68.2	2581.5 ± 233.2
			
			(2623.9~3098.3)	(2479.3~2784.9)	(2386.6~2776.4)

CUS	12	W0	2904 ± 199.2	260.4 ± 45.0	2643.6 ± 210.7
			
			(2777.4~3030.6)	(231.8~289.0)	(2509.8~2777.4)
	
		W3	1489.3 ± 302.8**	107.5 ± 49.9**	1381.8 ± 483.4**
			
			(1296.9~1681.7)	(75.7~139.1)	(1074.8~1688.8)

Data are mean ± S.D. before (W0) and after (W3) CUS procedure. Data in bracket are 95% confidence intervals for each relevant statistic. **:*P *< 0.01 compared with week 0 by the same group

### Body and brain weights of foetuses

Body weight, but not brain weight, was higher for control foetuses than the body weight from the CUS group for both males and females (see Table 5).

**Table 5:** Body weight and brain weight of foetuses

Groups	n	Body weight (g)	Brain weight (mg)
Female foetuses of control	47	3.52 ± 0.10	166.3 ± 3.6
		
		(3.49~3.55)	(165.2~167.4)

Female foetuses of CUS	43	3.48 ± 0.16*	165.6 ± 3.0
		
		(3.43~3.53)	(164.7~166.5)

Male foetuses of control	40	3.67 ± 0.13	172.5 ± 2.6
		
		(3.63~3.71)	(171.7~173.3)

Male foetuses of CUS	49	3.61 ± 0.08*	170.6 ± 2.2
		
		(3.59~3.63)	(170.0~171.2)

Data are mean ± S.D. Data in bracket are 95% confidence intervals for each relevant statistic. *: *P *< 0.05, compared with control by the same sex

### Serum COR and CRH level in maternal rats and foetuses

Serum COR and CRH levels were significantly higher for maternal rats with CUS before pregnancy than for controls and were higher in foetuses in the CUS group than in the control group for both males and females (see Table 6).

**Table 6:** Serum COR and CRH levels of maternal rats and foetuses

Groups	n	COR (ng/ml)	CRH (pg/ml)
Maternal rats of control	8	139.5 ± 11.9	2.69 ± 0.19
		
		(129.5~149.5)	(2.53~2.85)

Maternal rats of CUS	12	202.5 ± 12.2**	3.63 ± 0.26**
		
		(194.7~210.3)	(3.46~3.80)

Female foetuses of control	47	174.2 ± 17.8	2.95 ± 0.51
		
		(169.0~179.4)	(2.80~3.10)

Female foetuses of CUS	43	220.3 ± 15.6**	3.76 ± 0.42**
		
		(215.5~225.1)	(3.63~3.89)

Male foetuses of control	40	158.8 ± 14.4	2.69 ± 0.40
		
		(154.2~163.4)	(2.56~2.82)

Male foetuses of CUS	49	191 ± 13.8**	3.6 ± 0.41**
		
		(187.0~195.0)	(3.48~3.72)

Data are mean ± S.D. Data in bracket are 95% confidence intervals for each relevant statistic. **: *P *< 0.01 compared with control and the data of foetuses compared with control by the same sex

### The ratio of 5-HIAA to 5-HT in the hippocampus and hypothalamus of foetuses

Table 7 shows levels of 5-HT higher in the hippocampus and hypothalamus of foetuses in the CUS group than in the control group, and 5-HIAA levels were lower in the hippocampus in foetuses in the CUS group than in foetuses in the control group. Levels of 5-HIAA in the hypothalamus did not differ between foetuses in the CUS group and control group for both males and females (see Table 7). The ratio of 5-HIAA to 5-HT was significantly lower for both male and female foetuses in the CUS group than in the control group for both brain areas (see Table 7).

**Table 7:** HPLC analysis results of 5-HT,5-HIAA and 5-HIAA/5-HT in the hippocampus and hypothalamus of foetuses

Groups	n	Position	5-HT (ng/g)	5-HIAA (ng/g)	5-HIAA/5-HT
Female foetuses of control	47	Hippocampus	215.4 ± 15.8	165.1 ± 9.6	0.771 ± 0.041
			
			(210.8~220.0)	(162.3~167.9)	(0.759~0.783)
		
		Hypothalamus	116.9 ± 8.2	44.5 ± 5.5	0.382 ± 0.021
			
			(114.5~119.3)	(42.9~46.1)	(0.376~0.388)

Female foetuses of CUS	43	Hippocampus	236.3 ± 16.1**	135.8 ± 15.7**	0.572 ± 0.059**
			
			(231.3~241.3)	(130.9~140.7)	(0.554~0.590)
		
		Hypothalamus	146.7 ± 13.8**	46.1 ± 3.3	0.313 ± 0.052**
			
			(142.5~150.9)	(45.1~47.2)	(0.297~0.329)

Male foetuses of control	40	Hippocampus	206.7 ± 16.4	173.2 ± 12.8	0.84 ± 0.069
			
			(201.4~212.0)	(169.1~177.3)	(0.818~0.862)
		
		Hypothalamus	141.6 ± 15.2	54.9 ± 3.8	0.391 ± 0.044
			
			(136.7~146.5)	(53.7~56.1)	(0.377~0.405)

Male foetuses of CUS	49	Hippocampus	238.5 ± 10.5**	149.3 ± 10.2**	0.633 ± 0.07**
			
			(235.5~241.5)	(146.4~152.2)	(0.613~0.653)
		
		Hypothalamus	175.3 ± 13.3**	53.2 ± 6.3	0.312 ± 0.042**
			
			(171.5~179.1)	(51.4~55.0)	(0.300~0.324)

Data are mean ± S.D. Data in bracket are 95% confidence intervals for each relevant statistic. **: *P *< 0.01 compared with control by the same sex and the same position

### 5-HT1A mRNA expression in the hippocampus and hypothalamus of foetuses

**Figure 1:**
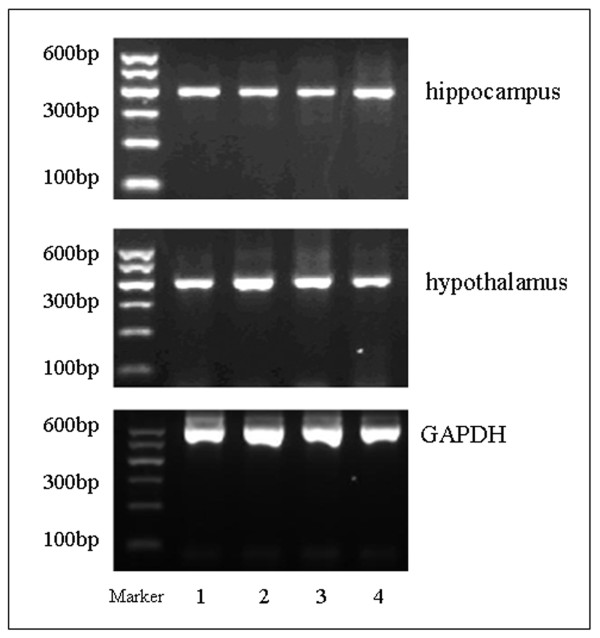
**RT-PCR analysis of 5-HT1A mRNA levels in the hippocampus and hypothalamus of foetuses**. Lanes 1,2: CUS females and males respectively; Lanes 3,4: control females and males, respectively; GADPH was a loading control.

Agarose gel electrophoresis revealed correctly sized 5-HT1A and GAPDH amplicons (Figure 1). The relative level of 5-HT1A to GAPDH was significantly lower in the hippocampus for both male and female foetuses in the CUS group than for foetuses in the controls, with no significant difference in the hypothalamus between foetal groups for either males or females (see Table 8).

**Table 8:** Results of 5-HT1A mRNA and SERT levels in the hippocampus and hypothalamus of foetuses

Groups	n	Position	5-HT1A mRNA(× 10^-2^)	SERT
Female foetuses of control	47	Hippocampus	0.284 ± 0.051	0.312 ± 0.069
			
			(0.269~0.299)	(0.292~0.332)
		
		Hypothalamus	0.272 ± 0.031	0.342 ± 0.059
			
			(0.263~0.281)	(0.325~0.359)

Female foetuses of CUS	43	Hippocampus	0.223 ± 0.036**	0.213 ± 0.06**
			
			(0.212~0.234)	(0.194~0.232)
		
		Hypothalamus	0.266 ± 0.059	0.246 ± 0.037**
			
			(0.248~0.284)	(0.234~0.258)

Male foetuses of control	40	Hippocampus	0.288 ± 0.051	0.334 ± 0.042
			
			(0.272~0.304)	(0.320~0.348)
		
		Hypothalamus	0.265 ± 0.044	0.351 ± 0.046
			
			(0.251~0.279)	(0.336~0.366)

Male foetuses of CUS	49	Hippocampus	0.233 ± 0.042**	0.226 ± 0.034**
			
			(0.221~0.245)	(0.216~0.236)
		
		Hypothalamus	0.275 ± 0.028	0.235 ± 0.055**
			
			(0.267~0.283)	(0.219~0.251)

Data are mean ± S.D. Data in bracket are 95% confidence intervals for each relevant statistic. 5-HT1A mRNA levels are the relative level of 5-HT1A to GAPDH. SERT levels are the relative level of SERT to β-actin. **: *P *< 0.01 compared with control by the same sex and the same position

### SERT expression in the hippocampus and hypothalamus of foetuses

**Figure 2:**
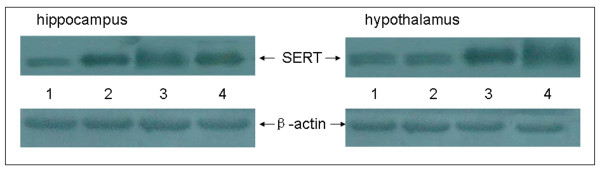
**Western blot analysis of relative SERT protein levels in the hippocampus and hypothalamus of foetuses**. Lanes 1,2: CUS females and males, respectively; Lanes 3,4: control females and males, respectively.

Signals of β-actin and SERT could be detected in the hippocampus and hypothalamus of foetuses (Figure 2). The relative level of SERT to β-actin as shown was lower in both the hippocampus and hypothalamus for foetuses in the CUS group than in the controls for both males and females (see Table 8).

### Correlations

Significant positive correlations were observed in serum COR as well as CRH levels between maternal rats and their foetuses (*r *= 0.674, *p *< 0.01; *r *= 0.709, *p *< 0.01, respectively). High COR and CRH levels in foetuses were correlated with low SERT expression in the hippocampus and hypothalamus (COR:*r *= -0.231, *p *< 0.05; *r *= -0.304, *p *< 0.01, respectively) (CRH: *r *= -0.378, *p *< 0.01; *r *= -0.359, *p *< 0.01, respectively) and with ratios of 5-HIAA to 5-HT (COR:*r *= -0.428 *p *< 0.01; *r *= -0.245, *p *< 0.05, respectively) (CRH:*r *= -0.491, *p *< 0.01; *r *= -0.263, *p *< 0.05, respectively). Low 5-HT1A expression in the hippocampus was highly correlated with a low ratio of 5-HIAA to 5-HT (*r *= 0.721, *p *< 0.01) and SERT expression (*r *= 0.683, *p *< 0.01).

## Discussion

We aimed to elucidate whether pre-gestational stress in maternal rats affects HPA axis activity and the serotonergic system in the brains of their foetuses. We examined 5-HT activity (5-HT, 5-HIAA and the ratio of 5-HIAA to 5-HT) and 5-HT1A receptor and SERT levels in brain regions of foetuses. The ratio of 5-HIAA to 5-HT and the level of SERT were significantly lower for foetuses in the CUS group than in the control group. Levels of 5-HT1A receptor were significantly lower in foetal hippocampuses in the CUS group than in the controls, with no significant difference in the hypothalamus. Finally, the level of SERT was lower in both the hippocampus and hypothalamus of foetuses in the CUS group than in the control group. The HPA axis and serotonergic system may be dysregulated in the foetuses of mothers exposed to CUS before pregnancy. From the results of this study, we extended our knowledge of pre-gestational stress-induced changes across several regions of the foetal brain and provided several novel findings about how pre-gestational stress can affect serotonergic systems in foetal rats with stressed mothers.

We successfully established a model of CUS in maternal rats. Body weight and behaviour findings for maternal rats generally agreed with our previous findings [3], so the CUS model was appropriate for our study.

Along with the reduced body weight of maternal rats with CUS, the reduced body weight of their foetuses suggests that growth retardation may relate to their mothers with CUS before pregnancy. However, brain weight did not differ between foetuses in the CUS group and control group. Therefore, the growth retardation of foetuses in the CUS group cannot explain all of our findings, which appears to relate to an imbalanced neuro-endocrine network after CUS in maternal rats and their foetuses.

The serum COR and CRH levels were higher in maternal rats with CUS before pregnancy than in control maternal rats. This finding may relate to the dysregulation of the HPA axis after exposure to chronic stress. This view is supported by previous studies [27,28]. We found that pre-gestational CUS in mothers significantly increased COR and CRH levels in foetuses in the CUS group as compared with controls for both males and females, and significant positive correlations in serum COR as well as CRH levels were observed between maternal rats and their foetuses in our study. Therefore, chronic stress can not only lead to an imbalance in the neuro-endocrine network of maternal rats, but it can also affect the HPA axis of the foetus via the maternal-placental-foetal interface [29]. Several studies have provided evidence that maternal stress causes elevated levels of circulating COR in the foetus [30]. The increase in the serum COR level of foetus results from reduced maternal COR-binding globulin and impaired regulation of the maternal HPA axis [31]. The HPA axis, impaired in maternal rats with CUS, may release stress hormones such as COR and CRH to enter the circulation of foetus directly through the placental barrier. On the other hand, the elevated level of circulating COR in maternal rats reduces the blood flow of the placenta, which decreases the supply of oxygen and nutrients to the foetus and results in a high level of COR in the foetus [29].

Early pregnancy is the most sensitive period in which stress is associated with neuro-behavioural changes in offspring [2]. Imposing CUS on maternal rats for a short time before pregnancy may also affect offspring in the early embryonic environment and thus have a profound influence on the brain development of offspring [3]. During the foetal lifespan, the brain undergoes rapid growth that is characterised by a high turnover of neuronal connections. Therefore, the foetal brain is especially vulnerable to hormones that may reach it in excess amounts from the maternal circulation as a result of stress [32]. Peptides such as CRH and beta-endorphin and COR may impede the formation of correct neural connections and reduce plasticity in the developing foetal brain [33]. Stress-induced levels of COR activate glucocorticoid receptors that are present in the rat hippocampus, hypothalamus, pituitary, cingulate cortex and amygdala from day 13 of gestation [34]. The ability of the foetal HPA axis to respond to maternal stress was shown by increased expression of CRH mRNA in the foetal para-ventricular nucleus (PVN) on day 15 of gestation in rats [35]. The abnormal HPA axis of the foetuses results in high levels of COR and CRH. High CRH levels affect γ-aminobutyric acid (GABA) levels and glutamate (Glu) neurons [36], thus leading to disturbed inhibitory amino acids (GABA) and excitatory amino acids (Glu), which may influence release of neurotransmitters such as 5-HT and down-regulate the sensitivity of 5-HT1A autoreceptors [37].

In this study, we showed that pre-gestational CUS induced decreased serotonergic activity in foetuses with decreased serotonergic ratios (5-HIAA to 5-HT) in the hippocampus and hypothalamus and decreased 5-HIAA levels in the hippocampus. Compared with control foetuses, both male and female foetuses in the CUS group showed increased 5-HT levels in the hippocampus and hypothalamus. Surprisingly, 5-HIAA levels were lower in the hippocampuses of foetuses in the CUS group than in control group, with no significant difference in levels in the hypothalamus of foetuses in the CUS group and controls for both males and females. Serotonergic ratio and serum COR and CRH levels in foetuses were significantly correlated. Decreased serotonergic status in the hippocampus and hypothalamus in foetuses in the CUS groups is probably directly or indirectly associated with changes in the HPA axis. By activating 5-HT1A receptors in the hippocampus, 5-HT causes hyper-polarisation [38], the suppression of hippocampal output and the subsequent disinhibition of HPA axis [39,40]. However, decreased serotonergic activity in foetuses in the CUS group could have resulted from changes in serotonergic function mediated by altered COR [41-43] and CRH [44,45] availability.

The 5-HT1A receptor is a crucial pre-synaptic autoreceptor in regulating the activity of 5-HT neurons and a post-synaptic receptor in the limbic system, cortex, hypothalamus and other parts of the brain [46]. Therefore, the regulation of 5-HT1A receptor can have an important role in psychological diseases. The hippocampus is a major limbic target of the brainstem serotonergic neurons that modulate emotion, cognitive and behaviour through post-synaptic 5-HT1A receptors [47]. Serotonergic neurons in the raphe nucleus are among the earliest to appear in the developing central nervous system. The hippocampus accepts the neurotransmission of 5-HT neurons from the dorsal raphe nucleus and median raphe nucleus [48]. The hippocampus also contains high levels of COR receptors in central brain areas. It is extremely vulnerable to excessive COR [49], which results in injury and may lead to decreased 5-HT1A receptor expression. Thus, pre-gestational stress may cause abnormal development of the serotonergic neurons in the hippocampus of foetuses by changing levels of stress hormones such as COR and CRH. We found lower 5-HT1A mRNA expression in the hippocampus of foetuses in the CUS group than in the control group and significant correlations between 5-HT1A and serum COR as well as CRH levels, which can support the above view. It appears that hippocampal serotonin is critical for programming of HPA function; however, it is possible that ascending fibres innervating the hypothalamus also play a role in this process. Many hypothalamic functions are regulated by the serotonergic system, which modulate the function of hypothalamus through dendrites of the 5-HT1A autoreceptor in cell bodies and the 5-HT1B autoreceptor at the nerve ends [50]. We found no difference between male or female foetuses in the CUS group and control group in level of 5-HT1A receptor in the hypothalamus, which suggests that the effect of pre-gestational stress on the hypothalamus might have a closer relationship with the 5-HT1B autoreceptor at the nerve ends.

We found a lower ratio of 5-HIAA to 5-HT and 5-HT1A mRNA expression in the hippocampus of both male and female foetuses in the CUS group than in the control group and lower levels of SERT in both the hippocampus and hypothalamus of foetuses in the CUS group, which suggests a possible link between the three factors. SERT is present throughout foetal development and is expressed at high levels at the time of neurogenesis and rapid brain growth. Serotonin levels within the synaptic cleft are regulated by the activity of the SERT protein, which actively re-uptakes serotonin into the pre-synaptic terminal and therefore regulates the duration of serotonin activity on its own autoreceptors [51]. In foetal rats, the main bilateral bundles of the ascending serotonergic axons pass through the mesencephalon, and a ventral branch leaves the medial forebrain bundle in the diencephalon to innervate the hypothalamus [52]. This pathway forms the direct synaptic connections between serotonergic neurons and the CRH-containing cells of the hypothalamic PVN [35]. In this connection, serotonin can activate HPA function through its excitatory effects on the PVN in adult animals, and the same likely occurs in the peri-natal period [53]. Our findings of the significant correlations between the SERT level and CRH levels in foetuses may support this view. In foetuses of the CUS group, which SERT was down-regulated in the hippocampus and hypothalamus, the serotonergic ratio (5-HIAA/5-HT) was also down-regulated in the same region. Indeed, SERT gene promoter variations conferred a blunted COR response in rhesus monkeys exposed to pre-natal stress [54]. Therefore, COR-mediated changes in SERT expression would likely have a significant impact on the metabolism of serotonin in the developing brain.

In our experiment, the impaired HPA axis of the foetuses of maternal rats exposed to CUS before pregnancy results in high levels of COR and CRH. High COR and CRH levels can alter serotonin-mediated neurotransmission and may directly affect serotonin levels pre-synaptically, in the synaptic cleft and post-synaptically [36,37]. Late pregnancy is known to be an important period for synaptic formation of serotonergic neurons in rats [55]. Pre-natal exposure to dexamethasone during late pregnancy in rats decreases serotonin turnover in the cerebral cortex, hypothalamus and hippocampus [56]. Exposure to COR during pregnancy leads to developmental changes in central serotonergic systems, as well as other brain monoaminergic systems, including the noradrenergic and dopaminergic pathways [57]. As serotonergic functions can be modified by COR and CRH, programmed changes in HPA function that are independent of serotonin will have a permanent effect on the serotonergic system. However, the relationship between dopamine (DA) or norepinephrine (NE) and 5-HT systems in the regulation of HPA axis activity are complex and not fully understood, and this requires further elucidation in our future experimental studies.

## Conclusions

In summary, three aspects of association likely exist between the 5-HT system and HPA-axis function in the foetuses of maternal rats with CUS before pregnancy: (1) Changes in serum COR and CRH levels during critical windows of development will lead to modifications in 5-HT activity (5-HT, 5-HIAA and ratio of 5-HIAA to 5-HT) and 5-HT1A receptor and SERT levels in the hippocampus and/or hypothalamus of foetuses. (2) Since serotonin is intimately involved in the tonic regulation of the CRH neuron, permanent developmental modification of the ascending serotonin system will lead to long-term changes in HPA function. (3) As serotonergic function can be modified by COR and CRH, programmed changes in HPA function that are independent of serotonin will have a permanent effect on the serotonergic system. Although pre-natal brain development is based on a genetic blueprint, pre-gestational stress can also have a profound influence. However, many questions still need to be answered, including those pertaining to the effects pre-gestational stress on the foetuses in the role of the 5-HT system in the regulation of HPA axis activity relate to other neurotransmitters such as DA and NE, sex differences on the response of HPA axis to stressful conditions, the specific signal transduction pathways and the effects of medical intervention. We will address these questions in our forthcoming studies.

## Abbreviations

COR: Corticosterone; CUS: Chronic unpredictable stress; CRH: Corticotrophin-releasing hormone; DA: Dopamine; E: Embryonic; EDTA: Ethylenediaminetetraacetic acid; GABA: γ-aminobutyric acid; GC: Glucocorticoid; GR: Glucocorticoid receptor; Glu: Glutamate; HPA: Hypothalamic-pituitary-adrenal; HPLC: High-performance liquid chromatography; NE: Norepinephrine; PVN: Paraventricular nucleus; SERT: Serotonin transporter; 5-HIAA: 5-hydroxy indole acetic acid; 5-HT: 5-hydroxytryptamine; 5-HT1A: 5-hydroxytryptamine 1A.

## Competing interests

The authors declare that they have no competing interests.

## Authors' contributions

HYJ and XHW are co-first authors, and they made equal contributions to this work. HYJ performed RT-PCR and the Western blot analysis and helped to draft the manuscript. XHW carried out the radioimmunoassay and the HPLC procedure and drafted the manuscript. LH participated in designing the experimental animal model. YHH performed the statistical analysis. SXC and CYB are co-corresponding authors, and they made equal contributions to this work. SXC conceived of the study and participated in its design. CYB participated in the overall design of the study. All authors read and approved the final manuscript.
